# Vertebral compression fractures: Still an unpredictable aspect of osteoporosis

**DOI:** 10.3906/sag-2005-315

**Published:** 2021-04-30

**Authors:** Fatma Yeşim KUTSAL, Gizem Olgu ERGİN ERGANİ

**Affiliations:** 1 Department of Physical Medicine and Rehabilitation, Faculty of Medicine, Hacettepe University, Ankara Turkey

**Keywords:** Osteoporosis, spine, spinal fractures, bone density

## Abstract

Vertebral compression fracture is a hallmark of osteoporosis (OP) and by far the most prevalent fragility fracture. It is well proven that patients who develop a vertebral compression fracture are at substantial risk for additional fractures. Diagnosis is based on adequate clinical evaluation, imaging, and laboratory tests. The imaging of OP and fragility fractures includes conventional radiology to evaluate spinal fractures, bone mineral density (BMD) testing by dual energy x-ray densitometry, quantitative computerized tomography, magnetic resonance imaging, bone scintigraphy (if necessary), and ultrasound. Screening and treatment of individuals with high risk of osteoporotic fracture are cost-effective, but approximately two-thirds of the vertebral compression fractures (VCF) that occur each year are not accurately diagnosed and, therefore, not treated. Evaluation of VCFs, even though they may be asymptomatic, seems essential to health-related and/or clinical research on OP.

## 1. Introduction

Osteoporosis (OP) is one of the most frequent metabolic bone disorders worldwide. It has been defined as a skeletal disorder characterized by compromised bone strength, predisposing a person to increased risk of fracture. OP is a silent disorder that does not display any evidence of disease until a fracture occurs. The health consequences of osteoporotic fractures not only have a negative impact on the quality of life but cause disability, as well. A vertebral compression fracture (VCF) is by far the most prevalent fragility fracture and is a hallmark of OP. It has been proven that patients who already have a VCF are at substantial risk for additional fractures [1–3]. One can say that VCF status is a powerful and independent risk factor for all new osteoporotic fractures, which is a major health care problem in the aging population since the incidence of these fractures increases with age [4]. 

Independently of bone mineral density (BMD) measurements, the prevalence and severity of VCFs have been shown to be predictive for the risk of new osteoporotic fractures [5]. 

If a VCF exists, the focus shifts to rehabilitation and prevention of the next fracture. These fractures can be linked with various problems such as back pain, sleeping problems, decreased activity, more bone loss, increased fracture risk, spinal deformity, decreased lung capacity, impaired function, increased comorbidities, and eventually mortality [6].

Although the concept of risk factor evaluation is gaining ground, the current clinical practice of OP assessment is still largely based on the evaluation of BMD. This is the main reason why most patients with VCFs are not clinically recognized. Additional imaging studies of the spine have not become routine for several reasons, including lack of awareness of VCF status as an independent risk factor and possibly because OP is a disease secondary to many other health problems; it is also not the “core” expertise of many physicians [7].

## 2. Epidemiology 

A diagnosis of OP or previous fragility fracture was reported in around one-third of patients by Ong et al. Most patients (75% male and 78% female) had 5 or more copathologies, and many of them were more dependent on activities of daily living on discharge compared to their preadmission level [8]. The incidence of new VCFs in females and males aged 50 years and over was 10.7/1000 people and 5.7/1000 people, respectively; the prevalence increased from 3% in females under 60 years of age to 20% in females over 70 years old and from 7.5 to 20% in males over the same age range [9].

Epidemiologic data related to osteoporotic fractures are limited in Turkey. In a retrospective chart review of 934 osteoporotic women, the aim was to explore the frequency of osteoporotic fractures in osteoporotic women on the basis of an outpatient clinic data and define the relationship between osteoporotic fractures and age, menopause status, BMD, and body mass index (BMI). Osteoporotic fractures were observed in 194 patients (20.8%). Vertebral compression fractures were the most common form of osteoporotic fracture (107 patients). The authors stated that there was no significant difference in terms of BMI between the patients with or without fractures [10]. 

As a matter of fact, most of the men with OP and osteoporotic fractures are not diagnosed and do not receive treatment. A crosssectional study included 2 groups of male patients: a total of 71 nursing home residents with a mean age of 76.0 years (nursing home group) and 44 men living in their homes with a mean age of 74.4 years (control group). BMD measurements were performed in all subjects, and the Spinal Deformity Index and Fracture Risk Assessment Tool were also used. OP was detected in 25.3% of patients in the nursing home group and 8.8% of patients in the control group. The authors stated that silent VCF was present in 27.8% of males older than 65 years. The VCF rate was higher in nursing home residents (42.2%) than in the control group (17.6%); in addition, male nursing home residents seemed at a higher risk for both OP and VCF. Results also showed that 5.6% of patients in the nursing home group and 8.9% of those in the male control group were aware of their VCFs [11].

## 3. Clinical manifestations

VCF is defined as a decrease of at least 15% to 20% in height of the vertebra. These fractures can occur anywhere in the spine, most commonly in the lower thoracic spine and due to minor activity such as coughing or getting in or out of the bathtub (for people with advanced OP). The majority of the compressive damage is limited to the front of the vertebral column, and the fracture is usually stable, so it can be rarely associated with nerve root irritation or spinal cord damage [8].

It is difficult to determine the cause and the exact time of fragility fractures of the vertebral body, and they often go undiagnosed. During evaluation of the patient, there are some clinical history details that can suggest a possible VCF. These include: (i) recent direct or indirect trauma, (ii) age, (iii) prolonged use of glucocorticoids, (iv) structural spinal deformity, and (v) loss of height > 6 cm. Therefore, it is strongly recommended to carefully evaluate the presence of dorsolumbar pain, progressive loss of height, or dorsal kyphosis. Multiple VCFs may result in alterations of some system functions, mainly pulmonary or gastrointestinal [12]. 

### 3.1. Symptomatology

The basic symptoms of a VCF are a sudden onset of back pain, which gets worse by standing or walking. Lying on one’s back makes the pain less intense. This is followed by limited spinal mobility, height loss, deformity, and disability. Some patients with VCFs report that they feel no back pain or other symptoms. Even if there is no back pain, middle-aged or elderly individuals (especially women) need to be concerned about potential fractures if there is evidence of any of the following: height loss, limited ability to twist and bend the back, or deformity that develops in the spine. The pain from an osteoporotic VCF typically lasts about 4 to 6 weeks as the bone heals. Some patients have reported that the more severe pain subsides and turns into more of a chronic, achy pain concentrated in the area of the back where the fracture occurred. This is because of the ligament problems due to postural changes. Musculoskeletal pain is common in elderly people, and clinical or subclinical VCFs are common causes. This pain may eventually result in functional and psychological impairments. Thorough physical examination is important in revealing the underlying cause of “pain.” [6,13] 

To identify individuals with asymptomatic VCFs, several clinical thresholds for height loss have been proposed. A 15° increase in kyphosis is associated with the presence of a VCF, but an adjustment for age should be done. Also, clinicians should keep in mind that it is important to demonstrate whether simple self-reported kyphosis is associated with the presence of VCFs determined by lateral radiographs since it is likely that patients with undiagnosed VCFs may feel the presence of kyphosis themselves. A crosssectional survey that aimed to clarify the associations of self-reported height loss and kyphosis with VCFs enrolled 407 women aged 60–92 years old who visited an orthopedic clinic in Japan. Kamimura et al. noted that both self-reported kyphosis and height loss were significantly associated with the presence and number of VCFs. As a result, these simple self-reports may be a useful tool for identifying undetected VCFs [3]. 

### 3.2. Impact on quality of life

Physical, emotional, and psychological incapacity, combined with the pain that results from hip, spine, or wrist fractures, can alter quality of life (QoL). QoL in men and women with OP should be thoroughly investigated, even prior to the occurrence of a fracture to develop appropriate interventions that can empower patients to effectively manage all stages of the disease [14,15]. It has been reported that VCFs have a negative impact on QoL, and their presence is linked with cardiopulmonary morbidities, depression, and death [6].

Numerous studies have documented the detrimental effect of fragility fractures on the health-related QoL (HRQoL) of individuals with OP [16,17]. In addition, researchers have agreed that an important marker of the clinical evolution of patients with OP and fractures is the assessment of HRQoL. Not only the fragility fractures but physical, emotional, and psychological incapacity can alter QoL [18–20].

A population-based crosssectional study which aimed to examine the association between prevalent VCF and back pain, neck pain, and HRQoL in elderly women and men, and which looked at possible sex-related differences in reported pain and HRQoL, included a total of 2887 individuals (1681 of whom were women) at a mean age of 65.4 years old. The study showed that prevalent VCF is associated with an increased risk of back pain and reduced HRQoL in postmenopausal women but not in men [21]. According to Salaffi et al., HRQoL scores were lower in women with lumbar VCFs compared with women with thoracic VCFs, only when the physical functioning and bodily pain dimensions approached statistical significance [22]. The number of VCFs was shown to be a determinant of a low QoL. As VCFs are usually asymptomatic and associated with reduced QoL, increased morbidity and mortality and an increased risk of future vertebral and nonvertebral fractures, detection remains an important challenge for clinicians [23]. 

## 4. Diagnostic approach

According to the literature, as many as one-third of all VCFs are never clinically diagnosed, mainly because of methodological problems. VCFs may also be asymptomatic, but it has been documented in several studies that osteoporotic VCFs may be associated with acute/chronic back pain [21]. The first step in the diagnosis is based on risk assesment.

### 4.1. Assessments of risks

Assessments of VCF risks are based not only on physical examination but also on a complete case history, laboratory, and diagnostic imaging tests. Complete case histories require additional information related to patients’ medical histories (especially the presence of comorbidities, any medication that may interfere with bone metabolism, previous fragility fractures, family history of fractures, gynecological history and age at the onset of menopause (in women)), lifestyle, and an evaluation of personal and environmental risk factors. According to the “guidelines for the management of OP and fragility fractures,” evaluation of the patient’s posture is mandatory, especially if there is an increase in kyphosis or height loss, which may indicate the presence of one or more VCFs [12].

Fracture risk assessment tool-FRAX can be used in clinical practice. This is a computer-based algorithm that permits the classification of risk. It has been documented that WHO FRAX algorithms have facilitated the assessment of fracture risk on the basis of fracture probability [24].

Diagnosis is based on adequate clinical evaluation, imaging, and laboratory tests; the accurate diagnosis of VCF is important for the treatment of OP and for the prevention of new fractures. Since many VCFs are asymptomatic or cause mild pain, the majority of VCFs are not diagnosed worldwide. Only 1 in 3 VCFs is clinically diagnosed and, according to the available data, the majority of cases are either undetected or incidentally detected by radiographic testing. 

Diagnostic imaging of OP and of fragility fractures includes basic conventional radiology to evaluate spinal fractures, BMD testing by DXA, quantitative computerized tomography (QCT), magnetic resonance imaging (MRI), bone scintigraphy (if necessary), and ultrasound (QUS). QCT, MRI, and bone scintigraphy are used for differential diagnosis. In symptomatic osteoporotic patients, bone scintigraphy can be helpful in elucidating the etiology of back pain. If central DXA is unavailable, QUS can be used to identify subjects at low or high risk of osteoporotic fracture.

In spite of the fact that the BMD assay is considered the best predictor of osteoporotic fragility fracture risks, it is always recommended that an adequate clinical evaluation be performed [12].

Jager et al. showed that combined vertebral fracture assessment (VFA) and the BMD method detect previously unknown VCFs in nearly 1 out of every 6 patients with a significant impact on management [7].

### 4.2. Radiology

VCFs need radiological confirmation (the semiquantitative method of Genant with conventional spine radiography is traditionally used in the evaluation of VCFs) but are often undiagnosed by radiologists, with a misdiagnosis rate of up to 50% [9]. The reasons for this inadequacy are the following: VCFs frequently do not present as a clinically recognizable event and many radiologically apparent VCFs go unreported [25,26].

### 4.3. Indications for vertebral radiographs

In a clinical practice guideline, Camacho et al. stated that if prevalent VCFs could alter clinical management for patients with unexplained height loss or back pain, thoracic and lumbar spine radiography or VFA by DXA is indicated. The sensitivity for detecting prevalent VCFs seems low, but these height loss thresholds have >90% specificity. Also, if there is kyphosis or systemic glucocorticoid therapy, vertebral radiographs are indicated [27].

### 4.4. Reporting vertebral fractures

When reporting VCFs, radiologists and clinicians should avoid using ambiguous terms such as “collapse,” “compression,” “loss of height,” “wedging,” or “wedge deformity.” Instead, the terms “mild,” “moderate,” or “severe” to describe VCFs are recommended [25,26].

Radiographic studies have identified 3 types of VCFs: wedging (anterior), biconcavity (middle), and a total collapse of vertebra. These definitions depend on the type and severity of the spinal height reduction. For a more accurate identification, there are also 2 other methods. The first is the semiquantitative visual method, which is based on an initial phase of visual evaluation of images for differential diagnosis. This gradation of osteoporotic VCFs is called the Genant criteria and is classified as mild, moderate, or severe. The second one is the quantitative morphometric method, which is performed by conventional radiology or with DXA, using VFA software by lower radiation doses in a single image. The VFA technique is applied to assess the severity of the VCFs or to pinpoint a possible worsening of preexisting VCFs during follow-up [12]. 

### 4.5. Vertebral fracture assessment

Population-based assessment of VCFs can be carried out by common DXA densitometers. This method is VFA and has been used in many population settings. According to Waterloo et al., its sensitivity and specificity are comparable to vertebral radiographs in their ability to diagnose grade 2 (moderate) and grade 3 (severe) VCFs [21]. The VFA technique enables the acquisition of a patient-friendly alternative to conventional radiographs (with lower radiation exposure and relatively lower costs) for the assessment of VCFs in a one-stop diagnostic test. However, Malgo et al. stated that the advantage of lower-radiation doses used in certain BMD scanners can be associated with the drawback of poor image quality, which could lead to misclassification of VCFs for the ascertaining of a vertebra as nonevaluable, leading to an inaccurate estimation of fracture risk [5].

Considering the fact that that most osteoporotic VCFs are asymptomatic, it is difficult to identify symptomatic VCFs, especially in patients with concomitant fractures. Concomitant acute osteoporotic VCFs and previous VCFs are common and are often overlooked. Risk factors for the occurrence of concomitant acute osteoporotic VCFs are: a low T-score in DXA and the number of previous VCFs. Performing an MRI scan of the thoracic and lumbar spine with STIR and T1w sequences in patients with multiple acute osteoporotic VCFs or suspicion of concomitant acute osteoporotic VCFs can be useful in order to detect all acute concomitant VCFs and start adequate and effective fracture treatment [28].

## 5. Prevention and treatment

### 5.1. Fracture prevention

There are a number of unmet needs when assessing OP and a number of strategies to prevent the continual increase of the disease. These are: (i) optimizing peak bone mass in young adults, (ii) structural implementation of a four-step diagnostic procedure in patients with clinical risk factors for osteoporotic fractures: DXA, VFA, fall risk, and secondary OP, (iii) more adequate measurement of bone strength, (iv) reduction in the treatment gap, (v) new drugs with a better efficacy/safety profile, (vi) shared decision-making with optimal nonmedical and medical treatment (nonpharmacological interventions include specific physical exercises for OP to improve muscle strength and balance, decrease pain, and improve QoL), and (vii) new strategies such as treat to target and definition of high-risk patients [29].

Unfortunately, fracture prevention is suboptimal and the reasons are: (i) fractures do occur, mainly in the elderly, (ii) fear of severe side effects, (iii) lack of education in professionals and in the lay public, (iv) lack of engagement: OP is a low medical priority, (v) lack of coordination between health care systems, (vi) inadequate access to diagnostics such as BMD measurement and VFA, (vii) suboptimal predictive value of diagnostic techniques, (viii) the treatment gap, (ix) low adherence and compliance to antiosteoporotic drugs, (x) generic drugs, the nocebo-effect (negative counterpart); and (xi) lack of focus on muscle strength and fall prevention [29].

### 5.2. Treatment

Approximately two-thirds of the VCFs that occur each year are not accurately diagnosed and, therefore, not treated. The patients’ pain is often just thought of as back pain, resulting from “soft tissue injuries” or “spondylosis” or as a “common part of aging.” It should be kept in mind that despite the absence of VCFs, “bone resorption” due to OP may also cause back pain [6,13].

Since standardized and accepted treatment evidence-based concepts are missing for certain fracture types, the treatment of osteoporotic VCFs is widely empirical. As in other osteoporotic fractures in the elderly, the key for a good outcome may be a combination of interdisciplinary treatment approaches and adapted surgical procedures [30].

The basic treatment of vertebral fractures in the acute stage involves conservative measures such as bed rest, minor and major analgesic medications, physical therapy, and bracing. 

For all patients, optimizing vitamin D and calcium status, as well as recommendation of risk appropriate exercises and fall prevention stategies, are mandatory. According to the latest treatment algorithm published by Kanis et al., in addition to the categories of low and high risk espoused in the current IOF-ESCEO guideline, a very high-risk status can also be identified and is defined as a fracture probability that lies above the upper assessment threshold after a FRAX assessment. For women at high risk, treatment usually starts with an antiresorptive drug, while patients at very high risk usually need anabolic therapy followed by antiresorptive drugs [31]. 

In a recent clinical practice guideline, 4 principles were published for the management of OP and osteoporotic fractures: (i) country-specific assessment tools should be used to identify possible fracture risk, (ii) patient preferences should be included in treatment plans, (iii) all pharmacological treatments should be accompanied by nutritional and lifestyle changes and strategies for prevention of falls, and (iv) in postmenopausal women who are at risk, pharmacological treatments can reduce fracture rates with acceptable risk-benefit and safety profiles [32].

Pain due to vertebral fracture often lasts for 1–3 weeks and then begins to subside and disappears within a few months. However, in some cases, a biomechanical instability may develop and persist due to the severity and location of the VCF. Vertebroplasty or kyphoplasty may therefore be considered in patients with intractable pain. Potential risks associated with these procedures and uncertain benefits over the long term should be considered in these practices, and these interventions are not found to be suitable in patients with no symptoms or mild symptoms [12].  It is well known that in VCFs, the primary goal of the surgical approach is to stabilize the spinal column and correct the deformity. In order to guide clinical practice, several symptoms have been identified that are thought to be relatively specific indications for further investigation. This further examination is often reported to be MRI because it is the most preferred diagnostic modality. Vertebral augmentation has been recommended in patients with positive imaging results and also worsening of the symptoms (e.g., decreased vertebral heights, negative impacts on functioning, etc.) [33]. Nevertheless, regarding the role of kyphoplasty and vertebroplasty interventions, no definitive consensus has been reached. Also, no conclusions can be drawn about the superiority of cementoplasty techniques over conservative management, according to Longo et al [34].

## 6. Conclusion

It has been stated that the huge burden caused by OP-related fractures to individuals, healthcare systems, and societies should provide a clear impetus for the progression of such approaches [35]. The cost of these fractures is enormous and is forecast to steadily increase globally over the coming decades. Low BMD remains a key preventable risk factor for fractures. Screening and treatment of individuals with a high risk of fracture is cost-effective. Predictive tools including “clinical risk factors,” “minimization of falls risk,” and “public authorities’ support” to create Fracture Liaison Services are suggested as paramount strategies [36]. 

There is strong evidence and consensus about the disease and its complications, but physicians still do not put enough effort in the identification and prevention of osteoporotic VCFs. Evaluation of the VCFs, even though they may be asymptomatic, seems essential to health related and/or clinical researches on OP. It has been suggested that physicians should give much more attention to their research efforts in increasing the awareness of not only the clinicians but the public, as well. Recommendations for primary screening are being developed to reduce mortality and morbidity caused by fragility fractures [37]. These practices, which are becoming increasingly important in terms of the health policies of countries, should be reviewed not only from the vantage point of health but also in terms of social, psychological, and economical perspectives. 
